# Drug–drug interactions and clinical considerations with co-administration of antiretrovirals and psychotropic drugs

**DOI:** 10.1192/j.eurpsy.2022.1152

**Published:** 2022-09-01

**Authors:** S. Petrykiv, M. Arts, L. De Jonge

**Affiliations:** 1 GGZWNB, Psychiatry, Halsteren, Netherlands; 2 GGZWNB, Psychiatry, Bergen op Zoom, Netherlands; 3 Leonardo Scientific Research Institute, Neuropsychiatry, Bergen op Zoom, Netherlands

**Keywords:** HIV, psychotropic drugs, Side effects, Drug-drug interactions

## Abstract

**Introduction:**

Psychotropic medications are frequently co-prescribed with antiretroviral therapy (ART). Hepatic metabolism both of AP and ART involves the cytochrome P450 enzyme system, potentially leading to a multitude of pharmacokinetic (PK) interactions and serious adverse side effects. The magnitude and clinical impact of PK-interactions can vary significantly.

**Objectives:**

The scope of this review is to summarize the currently available data regarding drug-drug interactions (DDI) between AP and ART, and to provide recommendations for their management.

**Methods:**

A formal search of Embase, Cochrane and Medline was performed, searching for human studies from inception till 2017 on PK-interactions between AP and ART and reporting clinical toxicity as outcomes. Authors also provide their expertise on magnitude and clinical relevance of DDI using PK interaction chart.

**Results:**

Ten case reports including total of 13 patient were analyzed, comprising following AP: aripiprazole (N=2), risperidone (N=4), quetiapine (N=3) and lurasidone (N=1) in combination with various ART regiments. Significant PK-interactions were to occur in cases when aripiprazole was combined with ritonavir and/or cobicistat or efavirenz and/or darunavir; risperidone with indinavir of ritonavir; quetiapine with ritonavir and atazanavir/ritonavir; lurasidone with atazanavir. Adverse events occurred in combinations of aripiprazole with ritonavir/darunavir, risperidone with ritonavir or indinavir, quetiapine with atazanavir and lurasidone with atazanavir.

**Conclusions:**

Psychotropics and antiretrovirals may be used safely, particularly when known DDIs are proactively managed. Clinicians should be aware of the pharmacokinetic and pharmacodynamic properties of these agents to best direct therapy and to provide optimal patient care

**Disclosure:**

No significant relationships.

